# Modeling the spatial structure of the endemic mara (*Dolichotis patagonum*) across modified landscapes

**DOI:** 10.7717/peerj.6367

**Published:** 2019-02-12

**Authors:** Milagros Antún, Ricardo Baldi

**Affiliations:** 1 Grupo de Estudio de Mamíferos Terrestres, Instituto Patagónico para el Estudio de los Ecosistemas Continentales (IPEEC)–CONICET, Puerto Madryn, Argentina; 2 Wildlife Conservation Society, Buenos Aires, Argentina

**Keywords:** Distribution and abundance, *Dolichotis patagonum*, Natural and anthropic factors, spatial models, Patagonia, Península Valdés

## Abstract

Across modified landscapes, anthropic factors can affect habitat selection by animals and consequently their abundance and distribution patterns. The study of the spatial structure of wild populations is crucial to gain knowledge on species’ response to habitat quality, and a key for the design and implementation of conservation actions. This is particularly important for a low-density and widely distributed species such as the mara (*Dolichotis patagonum*), a large rodent endemic to Argentina across the Monte and Patagonian drylands where extensive sheep ranching predominates. We aimed to assess the spatial variation in the abundance of maras and to identify the natural and anthropic factors influencing the observed patterns in Península Valdés, a representative landscape of Patagonia. We conducted ground surveys during the austral autumn from 2015 to 2017. We built density surface models to account for the variation in mara abundance, and obtained a map of mara density at a resolution of four km^2^. We estimated an overall density of 0.93 maras.km^−2^ for the prediction area of 3,476 km^2^. The location of ranch buildings, indicators of human presence, had a strong positive effect on the abundance of maras, while the significant contribution of the geographic longitude suggested that mara density increases with higher rainfall. Although human presence favored mara abundance, presumably by providing protection against predators, it is likely that the association could bring negative consequences for maras and other species. The use of spatial models allowed us to provide the first estimate of mara abundance at a landscape scale and its spatial variation at a high resolution. Our approach can contribute to the assessment of mara population abundance and the factors shaping its spatial structure elsewhere across the species range, all crucial attributes to identify and prioritize conservation actions.

## Introduction

Habitat selection has been defined as the process by which individuals use or occupy a non-random set of available habitats, and depends on the particular requirements of a given species or population, the availability of resources and the ability of individuals to exploit those resources (Morris, 2003). In addition, the patterns of habitat selection influence population dynamics through differences in survival and breeding success across habitats types (Pulliam & Danielson, 1991). Thus, variation in habitat quality (i.e., the different combinations of physical and biotic conditions affecting individual fitness) will be reflected in the variation in population density (Bradshaw et al., 1995; Mayor et al., 2009). Across modified landscapes, both natural and human-related factors are known to influence the abundance and distribution of wild species, as changes imposed by human activities can favor or limit species’ presence and subsequently affect biodiversity at a given area (Hansson, Fahrig & Merriam, 1995). Therefore, reliable models accounting for the spatial variation in the abundance of wild populations are crucial to gain knowledge of the response of species to habitat quality and to predict the consequences of implementation of conservation actions (Fischer & Lindenmayer, 2006).

The Mara (*Dolichotis patagonum)* is a large caviomorph rodent endemic to Argentina, widely distributed across the arid lands of the Monte and Patagonian steppe ecoregions (Taber, 1987; Kufner & Chambouleyron, 1991; Campos, Tognelli & Ojeda, 2001). In Patagonia, pioneering work conducted by Taber (1987), Taber & Macdonald (1992a, 1992b) showed that maras are monogamous and breed communally, an unusual combination among mammals. Maras dig breeding dens in which the young remain until they are 6–8 weeks old (Taber & Macdonald, 1992a; Baldi, 2007). Female maras give birth and nurse their pups at the entrance of the dens. Adults never occupy the dens, and their home range can reach two km^2^ (Taber & Macdonald, 1992b). Maras have been defined as generalist herbivores as they feed on grasses and shrubs (Bonino et al., 1997; Campos, Tognelli & Ojeda, 2001; Sombra & Mangione, 2005). The antipredatory strategy, of the species is based on the early detection and escape from predators (Dubost & Genest, 1974; Taber & Macdonald, 1992a). Consequently, maras would be favored by habitats that offer good visibility and access to shelter, like flat, open areas with heterogeneous vegetation structure (i.e., the presence of shrubs). Although there are studies suggesting that open sites and the proximity to ranch buildings would favor the presence of maras, past research has been focused on the location of breeding warrens, in particular the occurrence of communal dens (Taber & Macdonald, 1992a; Baldi, 2007; Alonso Roldán & Baldi, 2016) and habitat use by individuals around the breeding sites (Taber & Macdonald, 1992b; Rodríguez, 2009; Alonso Roldán et al., 2017). Also, it has been suggested that overgrazing by livestock lead to the decrease in cover of palatable grasses and the increase of woody species and bare soil could affect habitat use by maras (Kufner & Chambouleyron, 1991; Taber & Macdonald, 1992b; Rodríguez, 2009). The mara has been assessed as a “Near Threatened” species by the International Union for the Conservation of Nature (IUCN; Roach, 2016), as its global population has been reported to be dwindling due to habitat loss. Although estimates of population abundance and distribution were identified as the main research priorities (Roach, 2016), the available estimates of abundance are restricted to particular dens surveyed intensively during the breeding season (Taber & Macdonald, 1992a; Baldi, 2007; Alonso Roldán, Bossio & Galván, 2015), while estimates of abundance and distribution at a population scale in relation to habitat variables are lacking.

Our aim in this work was to account for the spatial variation in the abundance of maras at a population scale in Península Valdés (PV), a representative area of the arid Patagonia where wild species share the range with human activities. We used density surface models (DSM, Miller et al., 2013) which combine survey methodologies with mathematical models to obtain reliable estimates of abundance, while identifying the main factors related to its spatial variation. We hypothesize that both natural and human-related variables shape the spatial variation in the abundance of maras throughout the area. We predict that higher plant productivity, heterogeneity in vegetation structure, and flat terrain will all positively affect the number of maras. Regarding human-related factors, the proximity to infrastructure such as ranch buildings will favor the occurrence of maras and affect their spatial structure at the population scale, while high sheep stocking rates are a disturbance which will result in decreased numbers of maras.

## Materials and Methods

The present work is a non-invasive study, conducted through the observation of animals by means of binoculars. Permission for the research was given by the Direction of Conservation and Protected Areas, and the Direction of Wildlife of the Province Chubut (DF & FS-SSG, Permits 71/2014, 73/2015, and 69/2016).

### Study site

**Figure 1:**
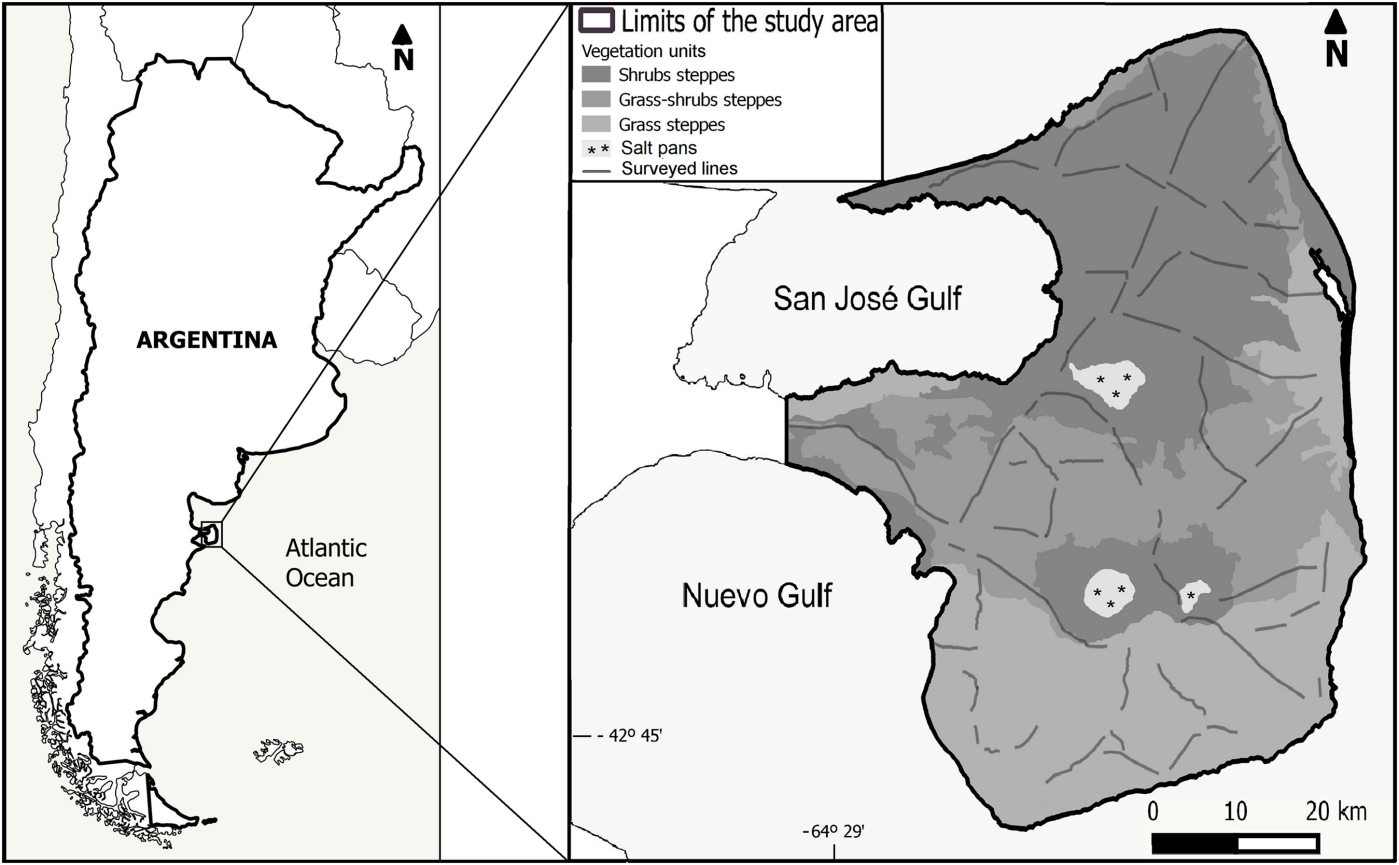
Location of the study area, distribution of the survey transects and vegetation units following Bertiller et al. (2017).

The study was conducted at PV, located in the Argentine Patagonia (Fig. 1), a provincial protected area and also a UNESCO World Heritage Site since 1999. The climate of PV is temperate semi-arid with a mean annual temperature of 13.6 °C, while annual precipitation averages 230 mm with a high interannual variation (Coronato, Pessacg & Alvarez, 2017). The vegetation is characteristic of the southern Monte Phytogeographic Province, but sharing plant species with the northern Patagonian Province (León et al., 1998). The vegetation structure is highly patchy, with high-cover vegetation, surrounded by areas with a high proportion of bare soil. The main life forms in PV are shrubs (evergreen and deciduous), bunch perennial grasses, and forbs (Sala et al., 1989; Golluscio & Sala, 1993; Bertiller et al., 2017). Shrubs and grass-shrubs steppes dominate northern and central PV with a vegetation cover that varies between 40% and 60%, while grass steppes predominate in the southern part of the area with an average cover of 70% (Fig. 1; Bertiller et al., 2017). The most common shrub species are *Chuquiraga avellanedae* and *Chuquiraga erinacea*, while the most abundant perennial grasses are *Nassella tenuis, Piptochaetium napostaence,* and *Sporobolus rigens* (Bertiller et al., 2017).

Extensive sheep ranching for wool production occupies most of the land, which is divided by fences into more than 60 properties. Each ranch is subsequently fenced into paddocks of 1,000–2,500 ha where the sheep graze on the native vegetation. There is usually one building per ranch permanently occupied by a rural worker, and occasionally an outstation which may be inhabited temporarily.

Species like the grison (*Galictis cuja*), Patagonian gray fox (*Lycalopex gymnocercus*) and the red-backed hawk (*Buteo polysoma*) have been reported as predators of the mara in Patagonia (Taber, 1987). Other potential predators of maras in PV are the puma (*Puma concolor*), the culpeo fox (*Lycalopex culpaeus*), and smaller cats (*Leopardus geoffroyi* and *Leopardus colocolo*; Nabte, 2010; Taber, 1987).

### Field surveys

We conducted ground, line transect surveys (Buckland et al., 1993; Laake et al., 1993) of maras during the austral autumn of 2015, 2016, and 2017 totaling 1,085.4 km surveyed along secondary dirt-roads and tracks, spaced by at least one km among contiguous tracks (Fig. 1). Surveys were conducted during the non-breeding period in order to maximize the number of observations, as maras tend to be aggregated around communal dens during the breeding season (Taber, 1987; Baldi, 2007); and to prevent possible biases in the abundance estimates due to pup mortality associated to the breeding season (estimated around 55% of the pups born between August and December; Baldi, 2007). All surveys were conducted from an open pickup truck, traveling at a maximum speed of 25 km.h^−1^, with two observers standing in the back. For every group of maras detected (one or more individuals) we stopped the vehicle, counted the number of animals using binoculars, estimated the perpendicular distance from the transect line to the location where the group was standing at the time it was detected, using a laser rangefinder (Bushnell Yardage Pro 1000; Bushnell Outdoor Products, Overland Park, KS, USA), and recorded our location and the angle relative to the group of animals using a portable GPS (Garmin Oregon 550; Garmin, Olathe, KS, USA).

### Estimating the detection function

Using standard distance sampling methodology (Buckland et al., 1993), we fitted a detection function *g(y)* to account for the probability of detecting maras. The detection model assumes that all groups were detected at zero distance from the transect line, with detectability decreasing with increasing distance from the line (Buckland et al., 2001). Following Thomas et al. (2010), we evaluated the half-normal, uniform, and hazard-rate functions as candidate detection functions. As the effect of data truncation (removal of the 5–10% of the sightings corresponding to the most extreme distance values; Thomas et al., 2010) increases robustness of the fit for the models, and that sightings far away from the line contribute little to fit the model at small distances (Buckland et al., 2001, 2015), we removed 10% of the sightings resulting in a truncation distance at 304 m from the transect line. Then, following Buckland et al. (2001) and Thomas et al. (2010), we visually explored frequency histograms of distances of each candidate function and selected the best model by the “*shape criterion,*” which is based on the analysis of the most critical region of the function close to the line, excluding functions that are spiked near zero distance. The detection function should have a “shoulder” close to the line, indicating that detection remains nearly certain at small distances (Buckland et al., 1993, 2001, 2004; Thomas et al., 2010; Supplemental Information 1). All analyses were performed using the “Distance” package version 0.9.7 (Miller, 2017) for R.

### Predictor selection

According to our hypotheses, we identified natural and anthropic variables as potential predictors of mara abundance (Table 1). Additionally, we included the geographic latitude and longitude as proxy variables to account for possible remaining variation (Table 1). Normalized Difference Vegetation Index (NDVI) from 250 m MODIS MOD13Q1 satellite images (available at https://lpdaac.usgs.gov) was used as a correlate of primary productivity. We calculated the mean values of NDVI for the spring-summer seasons (from September 21st to March 21st) of the years 2014–2015, 2015–2016, and 2016–2017 according to the field surveys. As some areas of PV are a mosaic of vegetation types, we found that a continuous variable such as the coefficient of variation (CV) of the mean NDVI values was better to represent changes in vegetation physiognomy than a categorical variable. Thus, we calculated the CV of the NDVI between 2010 and 2014 to account for variation in vegetation physiognomy, and found that it was larger across shrub steppes than in mixed and grass steppes (see Supplemental Information 2). Values of CV of altitude were obtained from the Digital Elevation Model for South America (resolution of about 220 m) at https://lta.cr.usgs.gov/SRTM1Arc. Updated numbers of sheep per paddock were obtained by asking owners and workers of the ranches during the field surveys. Data on the location of ranch buildings was available at our institute but it was also checked and updated in the field while working across PV between 2015 and 2017. We obtained the values for each variable using the QGIS Open Source Geographic Information System (QGIS Development Team, 2016) and packages rshape2 version 1.4.2 (Wickham, 2007), raster version 2.5.8 (Hijmans et al., 2016) and ggplot2 version 2.2.1 (Wickham & Chang, 2016; R software, version 3.2.1, R Development Core Team, 2015). The range of values of each variable across the study area was included as far as possible in the surveyed tracks. Multicollinearity in predictor variables could make difficult to separate the effects on the response variable and to compare alternative models (Lennon, 1999), so we evaluated the collinearity between pairs of covariates taking the values measured at each segment (see below, “*Density surface model”*). We considered two predictors not to be collinear when Pearson’s correlation coefficients were <0.7 (Block, Morrison & Scott, 1998). The variables CV of NDVI and geographic latitude showed collinearity (|*r*| > 0.7), thus we kept the former due to its ecological significance.

**Table 1:** List and description of all the variables proposed.

Variable type	Name of the variable	Description
Natural	Mean NDVI	Mean normalized difference vegetation index for the spring-summer seasons of 2014–2015, 2015–2016, and 2016–2017 according to each field survey. Used as a correlate of plant productivity
	CV NDVI	Coefficient of variation of NDVI from 2010 to 2014. Used as a correlate of vegetation physiognomy
	CV altitude	Coefficient of variation of mean altitude. Used to describe the topography of the terrain
Anthropic	Ranch dist.	Distance to the nearest ranch building in meters.
	Sheep stock.	Sheep stocking rate (sheep.km^−2^) obtained per paddock
Proxy	Longitude	Longitude projected into meters using Universal Transverse Mercator zone 20
	Latitude	Latitude projected into meters using Universal Transverse Mercator zone 20

### Density surface model

Following Miller et al. (2013) and DSM methodology, each transect line was divided into smaller segments of 1.8 km in length, totaling 603 segments. Subsequently, each observation was assigned to its segment according to its location. The size of the segment was defined according to the information available for the species (maras move on average 1.7 km per day-and its average home range is 1.93 km^2^, Taber & Macdonald, 1992b), the detection function and the length of the transects. Given that there were no covariates other than distance in the detection function, the probability of detection (*p*) was constant for all segments. Therefore, we estimated mara abundance per segment (*n*) by the “count method” (Hedley & Buckland, 2004). In this way, the number of maras seen in each segment was described by a generalized additive model (GAM; Wood, 2006) as the sum of smooth functions of uncorrelated predictor variables measured at the segment.

$$E({\hat n_j}) = \hat p\;{A_j}{\rm{exp}}\left[ {\;{{\rm{\beta }}_0} + \mathop \sum \limits_k \;{f_k}\;({z_{jk}})} \right]$$

Where *E*(*n̂_j_*) is the expected number of maras in the *j*th segment, *p̂* is the estimated probability of detection of maras, *A* is the segment area, *z_jk_* is the value of covariate *k* in segment *j*, while *f_k_* represents the smooth function of the spatial covariate *k* and β_0_ is an intercept term. We used restricted maximum likelihood for smoothness selection (Reiss & Ogden, 2009; Wood, 2011). The concurvity of the smoothing term (Wood, 2006) was evaluated before and after fitting the models (Miller et al., 2018) to guarantee that any smoothing term could be approximated by one or more of the other smoothing terms in the model. The concurvity measures were very small in all the models evaluated, suggesting negligible concurvity (Wood, 2006; available as Supplemental Information 3). Following Miller et al. (2013) we explored three response distributions including: Tweedie, negative binomial, and quasi-Poisson. The Tweedie distribution offers a flexible alternative to the others, in particular when the data contains a high proportion of zero values (Candy, 2004; Shono, 2008; Peel et al., 2012). For each distribution we built a “base model” considered all the covariates as univariate smooths. We performed the covariate selection in each base model by removing the non-significant covariates (with approximate *P* < 0.01; Marra & Wood, 2011) and included an additional penalty for each smoothing term, which allowed the degrees of freedom to fall below 1 (Wood, 2006; section 4.1.6; Wood, 2011). Thus, we obtained three models as final candidates (Table 2) and subsequently we selected the best-fit model based on the inspection of residual plots. Residual autocorrelation was checked by inspecting the correlogram, which showed the behavior of the correlation between segments at a series of lags. Models were fitted using the “dsm” package version 2.2.16 (Miller et al., 2018) for R.

**Table 2:** Density surface models tested.

Final models	Response distribution	Significant variables	Exp. Dev.	Ab.	SE	CV
A	Tweedie	s(ranch dist.) s(longitude)	15.9	3,261	494	0.15
B	Quasi-Poisson	s(ranch dist.) s(longitude)	24.1	3,195	357	0.11
C	Negative binomial	s(ranch dist.) s(longitude)	9.18	3,047	559	0.18

**Notes:**

The best fitting model selected is shaded.

Exp. Dev., percentage of explained deviance; Ab., total number of individuals of *D. patagonum* estimated for the study area; SE, standard error; CV, coefficient of variation.

### Abundance and variance estimation

We overlaid a grid of four km^2^ cells to our study area, obtaining a prediction area of 3,476 km^2^. We excluded those zones adjacent to the coastal limits of the area and also inside the salt pans as they represent marginal habitat of the study area that have not been surveyed. Based on the cell covariate values, we predicted the number of maras for each cell resulting from the selected DSM, and subsequently obtained an overall estimate of abundance for PV. Given that the detection function did not have covariates, we calculated the uncertainty associated with the estimation for each four km^2^ cell by using the variation propagation method (Williams et al., 2011). In this way, we included the uncertainty associated with both the detection function and the spatial model (GAM) in our estimates of the variance (Miller et al., 2013).

## Results

**Figure 2:**
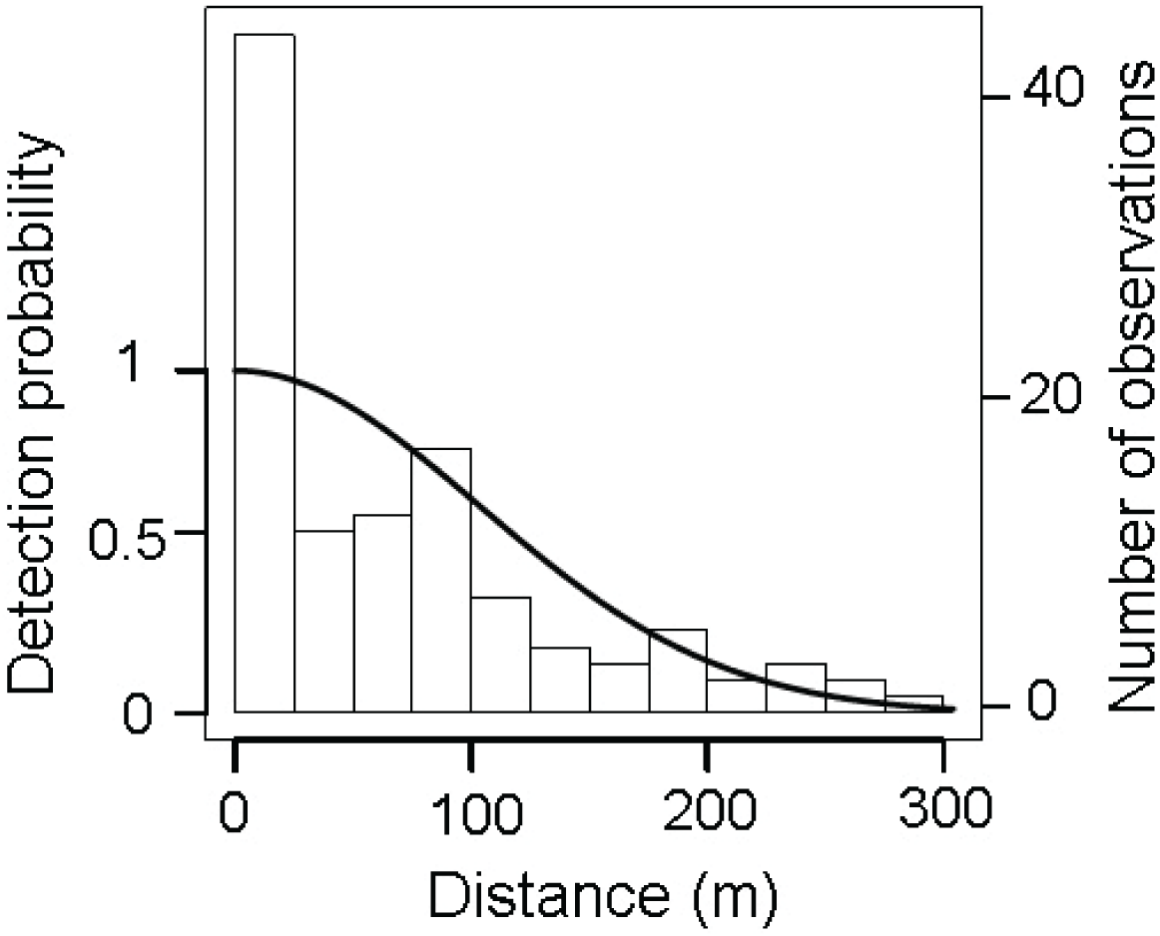
Distribution of perpendicular detection distances of *D. patagonum* sightings. Solid line represents the fitted half-normal detection function selected after the data truncation of the 10% of the most distant sightings. The bars represent the observed data grouped into distance intervals according to the perpendicular distance at which they were detected.

We recorded 119 sightings of maras (0.11 observations.km^−1^) comprising a total of 316 individuals, averaging 2.65 ± 1.76 individuals per observation (*X̂* ± SD). The detection function selected was the half normal (Fig. 2) with a truncation distance at 304 m from the transect line in order to remove the extreme 10% of the sightings and improve data fitting (Thomas et al., 2010). After truncation 107 sightings were retained, more than the minimum of 80 observations recommended for modeling clustered objects (Buckland et al., 2001).

**Figure 3:**
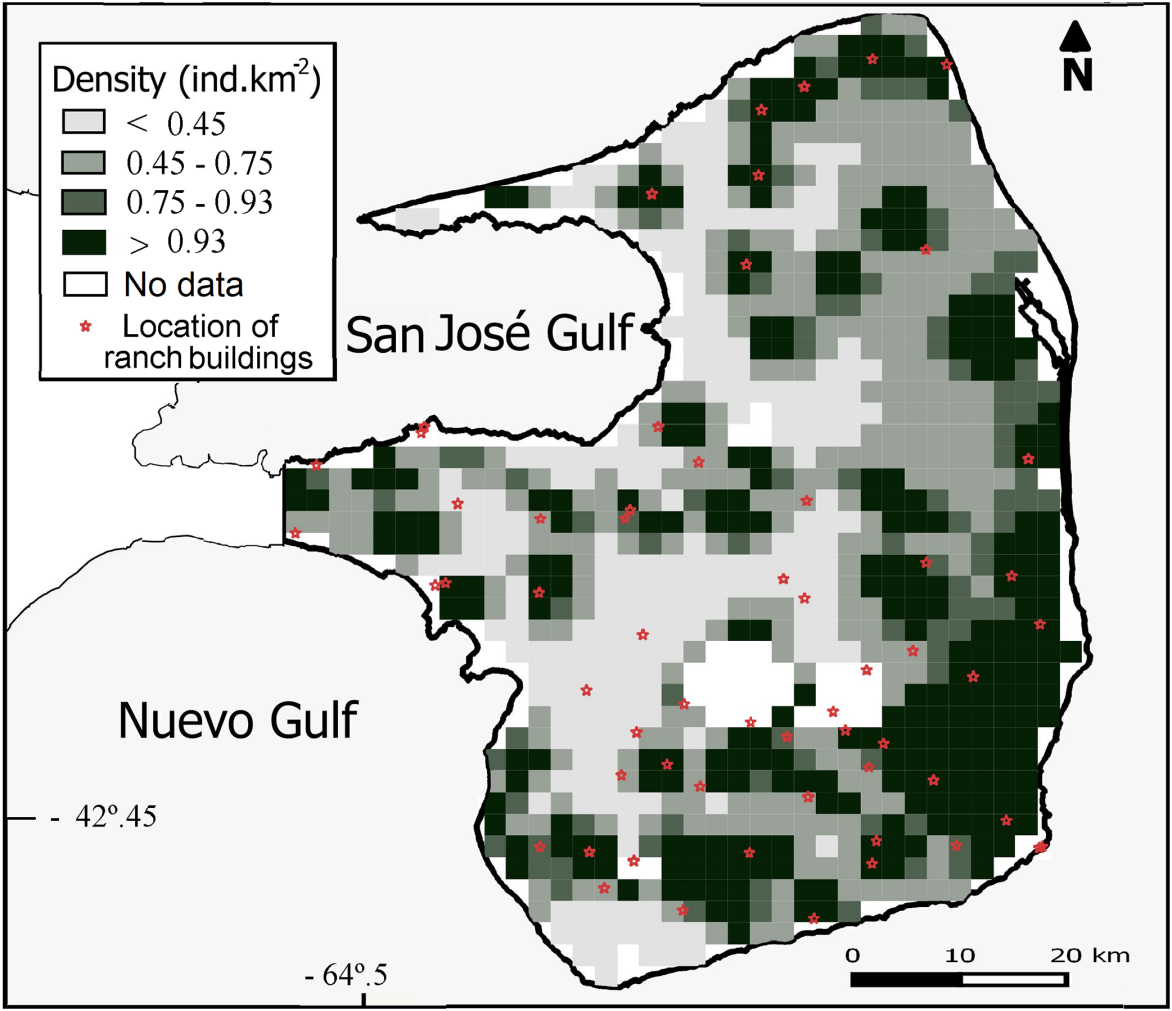
Spatial variation in the abundance of *D. patagonum*. Abundance is expressed in terms of absolute density (maras.km^−2^) for each four km^2^ cell, totaling a 3,476 km^2^ prediction area.

**Figure 4:**
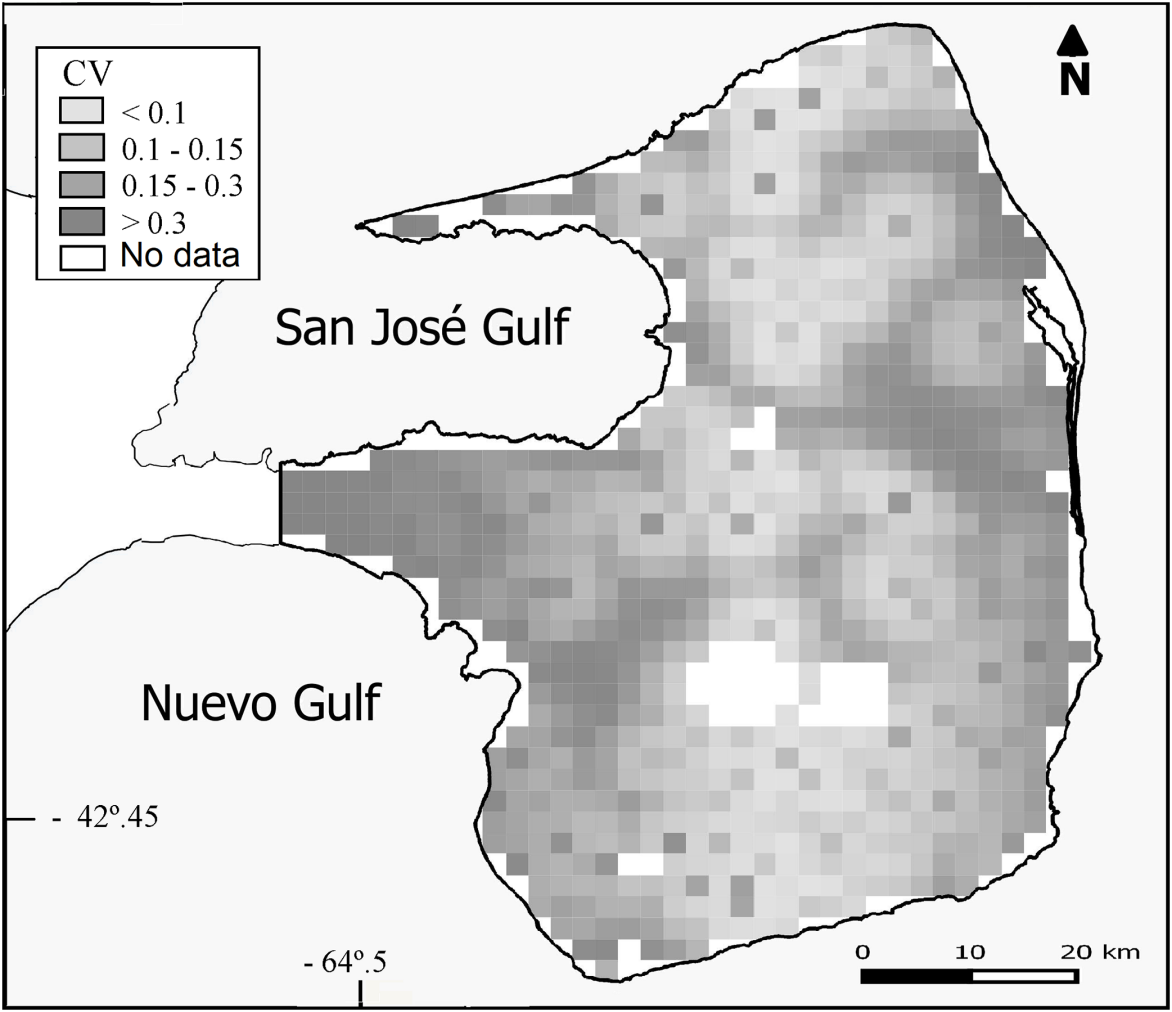
Uncertainty associated with the predicted abundance of *D. patagonum* per four km^2^ cell, in terms of the coefficient of variation (CV) of the estimate.

Overall population density estimated by the DSM was 0.93 maras.km^−2^ (CV = 15%; Table 2) for the 3,476 km^2^ prediction area (Fig. 3). Lowest densities (<0.45 ind.km^−2^) were mainly concentrated in the central and western areas of the Peninsula (Fig. 3), while the highest densities (>0.93 ind.km^−2^) were estimated for the eastern zone where ranch buildings tend to be more concentrated. The CV associated with the abundance estimation per cell showed a heterogeneous pattern (Fig. 4).

**Figure 5:**
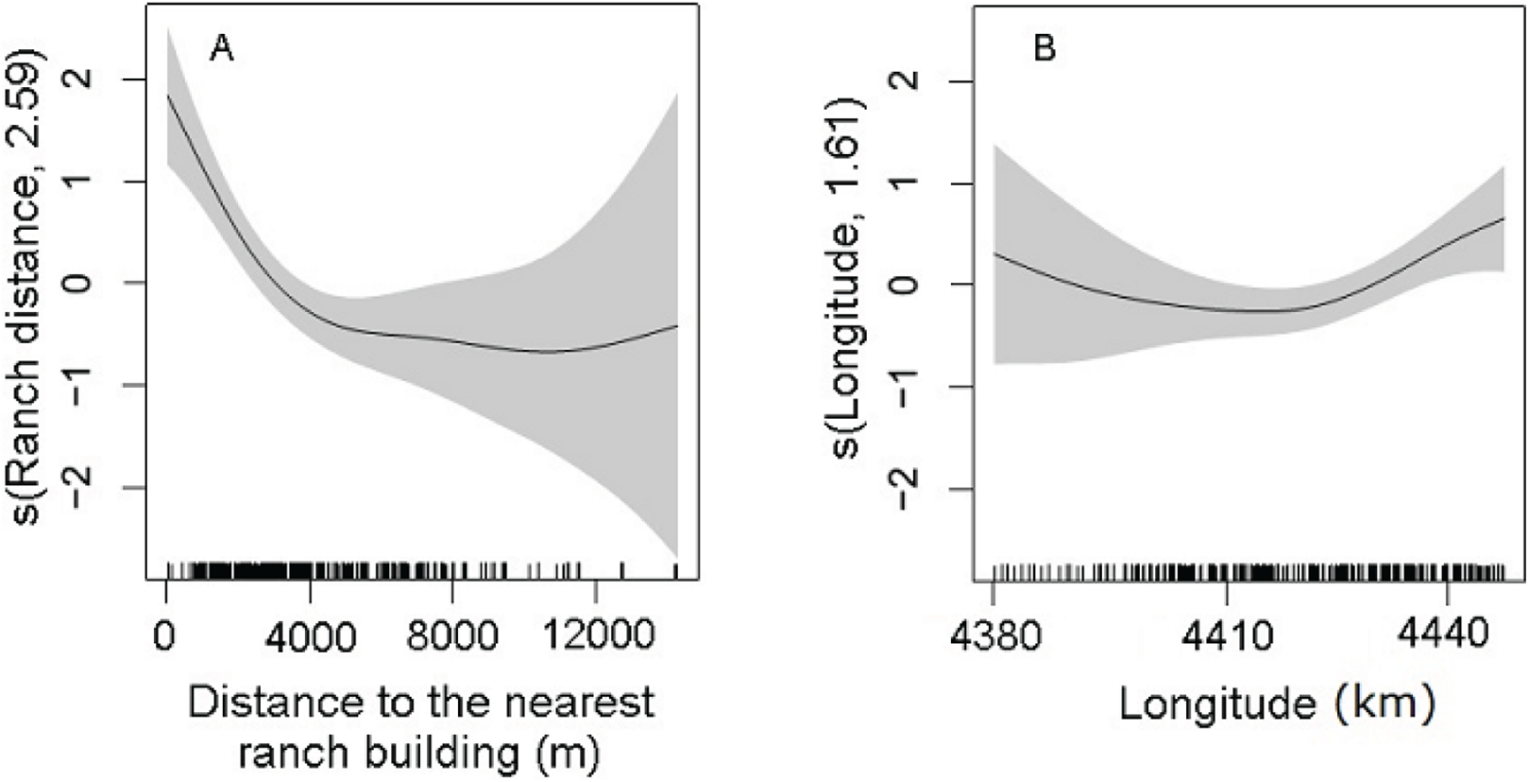
Partial effects of the significant predictors (A: Ranch distance; B: Longitude) on the abundance of *D. patagonum* according to the best fitting model. The solid lines represent the estimated smoothing terms (s) of each predictor and the gray shading the 95% confidence intervals for the mean effect. The number in brackets in each “s” gives the effective degrees of freedom (a measure of flexibility) of each term. The *y*-axis is on the scale of the link function. The tick marks at the bottom of the plot indicate the coverage of the range of values of each variable in the survey area.

Statistically significant variables (*P <* 0.01) of the selected DSM were the distance to the nearest ranch building (*P* = 5.96 × 10^−9^) and the geographic longitude (*P* = 0.001; Table 2). The abundance of maras had nonlinear relationship with the significant predictors. The confidence intervals of the smooth function of the predictor variables tended to be wider where the range of the variables had reduced survey coverage (Fig. 5). Maras were more abundant close to ranch buildings. Increased distance to the nearest ranch building showed a marked decrease in mara abundance, in particular within the range of 4,000 m (Fig. 5A). The geographic longitude showed a positive effect on the abundance of maras, from the central area of the PV to the eastern coast (Fig. 5B). A small amount of unmodeled correlation in residuals (<0.2) was observed between adjacent segments in the fitted model (see Supplemental Information 4), but we assumed that it did not affect the explanatory capacity of the model (Dellabianca et al., 2016).

## Discussion

Human dwellings are a key factor in mara’s habitat selection and strongly related to the species’ abundance in PV. Also, it is the only human-related factor explaining the spatial structure of the mara population. Although the main results do not contradict our hypothesis about natural and anthropic factors involved in mara habitat selection, only the geographic longitude could reflect some variation in environmental conditions, while predictive variables related to plant productivity, vegetation physiognomy and topography (mean NDVI, CV NDVI, and CV Altitude) did not show significant effects in the abundance of maras.

Human presence—represented by the distance to inhabited ranch buildings—favored the increase in mara abundance throughout the modified landscape of PV. Previous studies conducted at a local scale, focused on particular warrens during the breeding season, suggested that maras would gain protection from predators as the ranchers usually kill carnivores like the puma, gray and culpeo foxes, and smaller cats in order to protect their sheep (Taber & Macdonald, 1992a; Rodríguez, 2009; Rivas et al., 2015; Alonso Roldán & Baldi, 2016). Therefore, the proximity to inhabited ranch buildings could represent safe areas with low risk of predation for *D. patagonum* and likely this is reflected at a population scale.

It is known that human activity can alter the interactions between mammalian carnivores and their prey species (Berger et al., 2001; Schuette et al., 2013), leading to numerous consequences such as local irruptions of native and domestic herbivores (Sinclair, 1998), site-specific changes in prey behavior (Berger et al., 1999), and disease propagation (Wilson & Childs, 1997). For example, predator displacement by humans was found to result in a positive, indirect effect on prey species abundances such as elk (*Cervus elaphus*) and white-tailed deer (*Odocoileus virginianus*) in the drylands of southwestern Canada, where pumas and wolves (*Canis lupus*) are actively persecuted (Hebblewhite et al., 2005; Muhly et al., 2011). It is likely that human activities related to sheep ranching in PV are disruptive of predator-prey interactions and hence favor the local abundance of maras in the vicinity of ranch buildings. In Patagonia, carnivores are perceived by ranchers as a threat to their livestock (Sillero-Zubiri, Reynolds & Novaro, 2004; Travaini et al., 2000; Walker & Novaro, 2010), and this led to high hunting rates in areas frequented by humans or where the ranchers live (Novaro, Funes & Walker, 2005). As the mara is an important prey species across the Patagonian drylands (Walker & Novaro, 2010), different hunting pressure on carnivores resulting in differences in predation rates throughout the landscape (Novaro, Funes & Walker, 2005) could be reflected in mara distribution and abundance patterns. Also, it is likely that the abundance of maras is positively influenced by the availability of food in the vicinity of human dwellings. Usually, ranch buildings are close to temporary water bodies which provide single, resource-rich patches of nutritive food items where maras tend to feed all year round (Taber & Macdonald, 1992b). Therefore, lower predation risk and the higher food availability in areas close to human inhabitants could lead to an increase in the local abundance of maras. A positive effect of local high-density is the decrease of individual vigilance time and an increase of pup survival in communal warrens, as observed by Taber & Macdonald (1992a). Whilst density-dependent habitat selection and intraspecific competition are likely to play a role, further research is needed to understand the processes shaping the patterns of local abundance.

Although our work showed a positive effect of human presence on the abundance of maras, it is necessary to investigate what are the possible costs associated to this interaction. For example, there is evidence showing that maras are exposed to infectious disease like Johne’s disease and toxoplasmosis, common to the domestic sheep and the invasive European hare (*Lepus europaeus*) in PV (Marull et al., 2004). Therefore, the proximity to ranch buildings, which are next to shearing sheds and corrals were the sheep are gathered, could bring negative consequences for mara’s health. Regarding the abundance of sheep, we did not find effects on the abundance of maras in this study. It is known that livestock grazing and trampling drive changes in the vegetation structure (Van De Koppel, Rietkerk & Weissing, 1997; Bisigato & Bertiller, 1997; Bisigato et al., 2005) that subsequently affect the abundance and distribution of wild species (Longland & Young, 1995; Keesing, 1998; Campos, Tognelli & Ojeda, 2001; Tabeni & Ojeda, 2003). On the other hand, there is evidence that the diets of maras and sheep do not show a high overlap reducing the likelihood of competition for food resources (Bonino et al., 1986, 1997; Kufner & Pelliza De Sbriller, 1987; Campos, Tognelli & Ojeda, 2001; Sombra & Mangione, 2005; Rodríguez & Dacar, 2008). Nevertheless, specific studies designed to investigate mara-sheep interactions are needed to assess the effects of the abundant and widespread domestic species on the wild, low-density populations of maras.

Although the correlates of plant productivity and vegetation physiognomy had no significant effects on the spatial variation in the abundance of maras as we predicted, we cannot rule out their possible effects. It is likely that both the NDVI and its CV were not sufficiently sensitive variables to account for the variation in the composition of different life-forms affecting habitat selection by maras. However, the proxy variable “geographic longitude” did have a significant effect on the variation in mara abundance. Broadly, this variable could be interpreted as a good approximation to spatial variation in the rainfall regime, a crucial attribute controlling the presence and abundance of grasses and herbs across the arid systems (Noy-Meir, 1973). In PV, the average annual rainfall increases from the west toward the eastern coast (Coronato, Pessacg & Alvarez, 2017) where the model estimated the highest densities of maras (Fig. 3), and the relationship between mara abundance and geographic longitude was positive (Fig. 5B). This could be associated to the abundance of grasses and herbs which are important food items for the mara whose growth rates respond quickly to the rainfall regime (Kufner & Pelliza De Sbriller, 1987; Campos, 1997). The pre-breeding period of the mara occurs between May and August, when the precipitation tends to be higher and high-quality food items are more abundant. However, this study was limited to the post-reproductive period. Future research could incorporate the seasonal dynamics in abundance and distribution to analyze variation in habitat selection by maras throughout the year.

Using the DSM, we found maras occur at a low population density and they are positive related to human presence in PV, a protected area under managed resources (IUCN Category VI). Conservation authorities should consider the implementation of a monitoring program in order to evaluate population trends in the area, as well as the assessment of the factors affecting the abundance of maras in different management scenarios. As a Near-Threatened species reported to be declining, coordinate efforts are needed to expand population surveys and to identify the main threats to maras across their range.

## Conclusions

Natural and anthropic variables shape the spatial variation in the abundance of maras in PV. The location of ranch buildings was key in habitat selection by maras across the landscape, while the positive association between species’ abundance and geographic longitude could reflect the variation in the rainfall regime and ultimately in the abundance of grasses and herbs. Our results showed that maras are heterogeneously distributed and their population density is low across the modified landscape of PV, a representative area of the arid Patagonia. The use of density surface models allowed us to (i) obtain the first estimate of mara abundance at a population scale; (ii) describe its variation at a higher resolution; and (iii) identify the main variables explaining the spatial structure of the population. This approach can contribute to assess mara population abundance and distribution elsewhere across its range, by combining the well-known distance sampling survey method with spatial modeling. While the identification of the main variables explaining the variation in the abundance of maras is a first step toward the design of conservation actions, future research should focus on the mechanisms underlying the observed patterns and their effects on mara population dynamics.

## Supplemental Information

10.7717/peerj.6367/supp-1 Supplemental Information 1 Detection function modeling. As we mentioned in the subsection “*Estimating the detection function*,” we fitted a detection function *g(y)* to account for the probability of detecting maras by the standard distance sampling methodology (Buckland et al., 1993). We compared three different key functions as candidates, the half-normal, uniform and Hazard rate (Thomas et al., 2010). We removed the 10% of the sightings corresponding to the most extreme distance values (Buckland et al., 2001; Thomas et al., 2010; Buckland et al. 2015), resulting in data truncation set at 304 m from the line. Then, we visually explored frequency histograms of distances (Buckland et al., 2001; Fig. S1) and took into account the “*shape criterion*” to select the best model,. This criterion is based on the analyses of the most critical region of the function close to the line (Buckland et al., 1993; Buckland et al., 2001), being especially important where some data heaping at zero distance is suspected. Consequently, we decided to discard the hazard rate and uniform functions as the *shape criterion* suggests to exclude spiked functions near zero distance.

10.7717/peerj.6367/supp-2 Supplemental Information 2 Analysis of the variation coefficient of the Normalized Vegetation Index (CV NDVI). The variation coefficient of the Normalized Vegetation Index (CV NDVI) was calculated in each pixel–of 250 m resolution–from MODIS MOD13Q1 satellite images of the period 2010 to 2014, available at https://lpdaac.usgs.gov. The average values of sthe CV NDVI in each segment (1.8 × 2 km^2^ see in the main paper the section “*Density surface model (DSM)*”) and in each cell (4 km^2^) of the prediction grid (see in the main paper the section “*Abundance and variance estimation*”) were calculated. The map of the spatial variation of the CV NDVI was constructed (Fig. SI.1.1a) and the boundaries of the vegetation units of Península Valdés–defined by Bertiller et al. (2017; Fig. SI.1.1b)–were superimposed (Fig. SI.1.1a). Then, the mean NDVI CV was calculated in each vegetation unit (Table SI.1.1). The behavior of the variable in each stratum was visualized by the ‘box-plot’ chart (Fig. SI.1.2), while the significant differences were evaluated by means of Wilcoxon rank sum test (Table SI.1.1).

10.7717/peerj.6367/supp-3 Supplemental Information 3 Concurvity measures between smooth terms. As we described in the article, we evaluated concurvity measures between smooth terms throughout the model fitting procedure. Here we presented the pairwise concurvity measures by three related indices (worst, observed and estimated) for the base model of the Tweedie response distribution (Tables SI.2.1, SI.2.2 and SI.2.3), and for the final model selected (Tables SI.2.4, SI.2.5 and SI.2.6).

10.7717/peerj.6367/supp-4 Supplemental Information 4 Spatial autocorrelation in the residuals. Spatial autocorrelation in the residuals was evaluated using the ‘dsm.cor’ function of the‘dsm’ package. As described in the article, the correlogram show a small amount of spatial autocorrelation in the residuals (Fig. SI3.1). The confidence interval increased in width as the number of lags increased.

10.7717/peerj.6367/supp-5 Supplemental Information 5 Count data: number of observations, group size and segment coordinates.

10.7717/peerj.6367/supp-6 Supplemental Information 6 Modelling procedure. This file indicates the link that explained the modelling procedure that was implemented in this study.

10.7717/peerj.6367/supp-7 Supplemental Information 7 Segment data. Location and variables that define each segment.

## Additional Information and Declarations

### Field Study Permissions

The following information was supplied relating to field study approvals (i.e., approving body and any reference numbers): Permission for the research was given by the Direction of Conservation and Protected Areas, and the Direction of Wildlife of the Province Chubut (DF & FS-SSG, Permits 71/2014, 73/2015, and 69/2016).

### Data Availability

The following information was supplied regarding data availability: The raw data is available in Supplemental Files.
