# Supplementary material for: Modeling the spatial structure of the endemic mara (Dolichotis patagonum) across modified landscapes
Source: PeerJ. 2019 Feb 12;7:e6367. doi: 10.7717/peerj.6367 (PMC6376934; doi:10.7717/peerj.6367)
Supplement: Supplemental Information 1 — As we mentioned in the subsection “Estimating the detection function,” we fitted a detection function g(y) to account for the probability of detecting maras by the standard distance sampling methodology (Buckland et al., 1993). We compared three different key functions as candidates, the half-normal, uniform and Hazard rate (Thomas et al., 2010). We removed the 10% of the sightings corresponding to the most extreme distance values (Buckland et al., 2001; Thomas et al., 2010; Buckland et al. 2015), resulting in data truncation set at 304 m from the line. Then, we visually explored frequency histograms of distances (Buckland et al., 2001; Fig. S1) and took into account the “shape criterion” to select the best model,. This criterion is based on the analyses of the most critical region of the function close to the line (Buckland et al., 1993; Buckland et al., 2001), being especially important where some data heaping at zero distance is suspected. Consequently, we decided to discard the hazard rate and uniform functions as the shape criterion suggests to exclude spiked functions near zero distance. [file peerj-07-6367-s001.docx]

**Supplemental information SI1. Detection function modeling**

As we mentioned in the subsection “*Estimating the detection function*”, we fitted a detection function *g(y)* to account for the probability of detecting maras by the standard distance sampling methodology (Buckland et al., 1993). We compared three different key functions as candidates, the half-normal, uniform and Hazard rate (Thomas et al., 2010). We removed the 10% of the sightings corresponding to the most extreme distance values (Buckland et al., 2001; Thomas et al., 2010; Buckland *et al*. 2015), resulting in data truncation set at 304 m from the line. Then, we visually explored frequency histograms of distances (Buckland et al. 2001; Fig.S1) and took into account the “*shape criterion”* to select the best model,. This criterion is based on the analyses of the most critical region of the function close to the line (Buckland et al. 1993; Buckland et al., 2001), being especially important where some data heaping at zero distance is suspected. Consequently, we decided to discard the hazard rate and uniform functions as the *shape criterion* suggests to exclude spiked functions near zero distance.


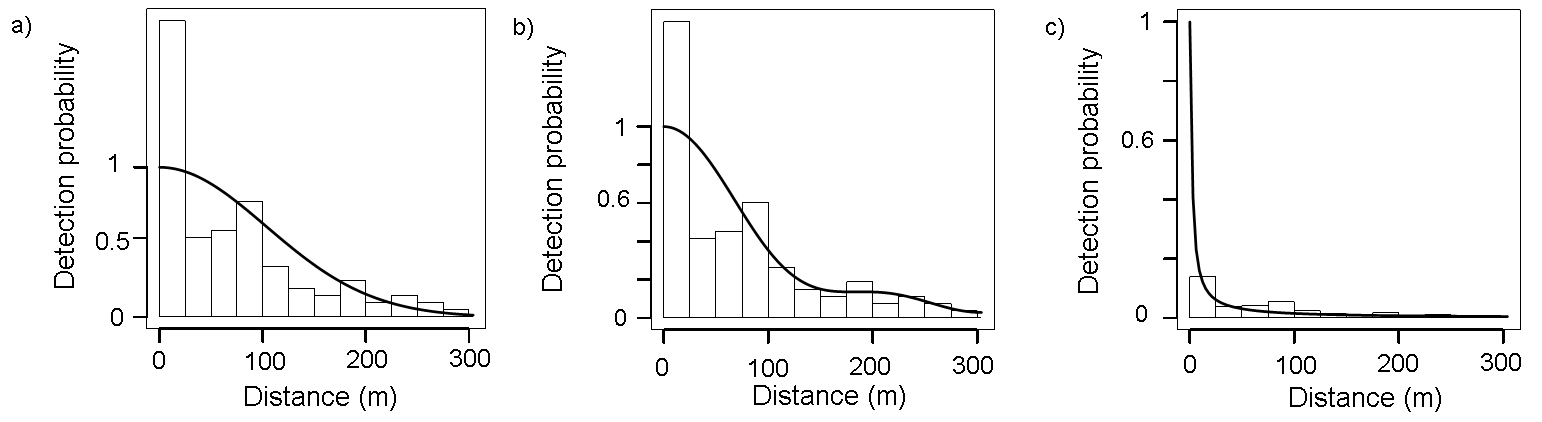


**Fig SI.1.** **Distribution of perpendicular detection distances of *D. patagonum* sightings.** Solid line represents the final fitted key functions: a) Half-normal, b) Uniform, c) Hazard rate. The bars represent the observed data grouped into distance intervals according to the perpendicular distance at which they were detected.
