# Supplementary material for: Modeling the spatial structure of the endemic mara (Dolichotis patagonum) across modified landscapes
Source: PeerJ. 2019 Feb 12;7:e6367. doi: 10.7717/peerj.6367 (PMC6376934; doi:10.7717/peerj.6367)
Supplement: Supplemental Information 2 — The variation coefficient of the Normalized Vegetation Index (CV NDVI) was calculated in each pixel–of 250 m resolution–from MODIS MOD13Q1 satellite images of the period 2010 to 2014, available at https://lpdaac.usgs.gov. The average values of sthe CV NDVI in each segment (1.8 × 2 km2 see in the main paper the section “Density surface model (DSM)”) and in each cell (4 km2) of the prediction grid (see in the main paper the section “Abundance and variance estimation”) were calculated. The map of the spatial variation of the CV NDVI was constructed (Fig. SI.1.1a) and the boundaries of the vegetation units of Península Valdés–defined by Bertiller et al. (2017; Fig. SI.1.1b)–were superimposed (Fig. SI.1.1a). Then, the mean NDVI CV was calculated in each vegetation unit (Table SI.1.1). The behavior of the variable in each stratum was visualized by the ‘box-plot’ chart (Fig. SI.1.2), while the significant differences were evaluated by means of Wilcoxon rank sum test (Table SI.1.1). [file peerj-07-6367-s002.docx]

**Supplemental information SI2. Analysis of the variation coefficient of the Normalized Vegetation Index (CV NDVI).**

The variation coefficient of the Normalized Vegetation Index (CV NDVI) was calculated in each pixel - of 250 m resolution - from MODIS MOD13Q1 satellite images of the period 2010 to 2014, available at https://lpdaac.usgs.gov. The average values of the CV NDVI in each segment (1.8 x 2 km^2^ see in the main paper the section “*Density surface model (DSM)”*) and in each cell (4 km^2^) of the prediction grid (see in the main paper the section “*Abundance and variance estimation*”) were calculated. The map of the spatial variation of the CV NDVI was constructed (Fig. SI.2.1a) and the boundaries of the vegetation units of Península Valdés - defined by Bertiller et al. (2017; Fig. SI.2.1b) - were superimposed (Fig. SI.2.1a). Then, the mean NDVI CV was calculated in each vegetation unit (Table SI.2.1). The behavior of the variable in each stratum was visualized by the 'box-plot' chart (Fig. SI.2.2), while the significant differences were evaluated by means of Wilcoxon rank sum test (Table SI.2.1).
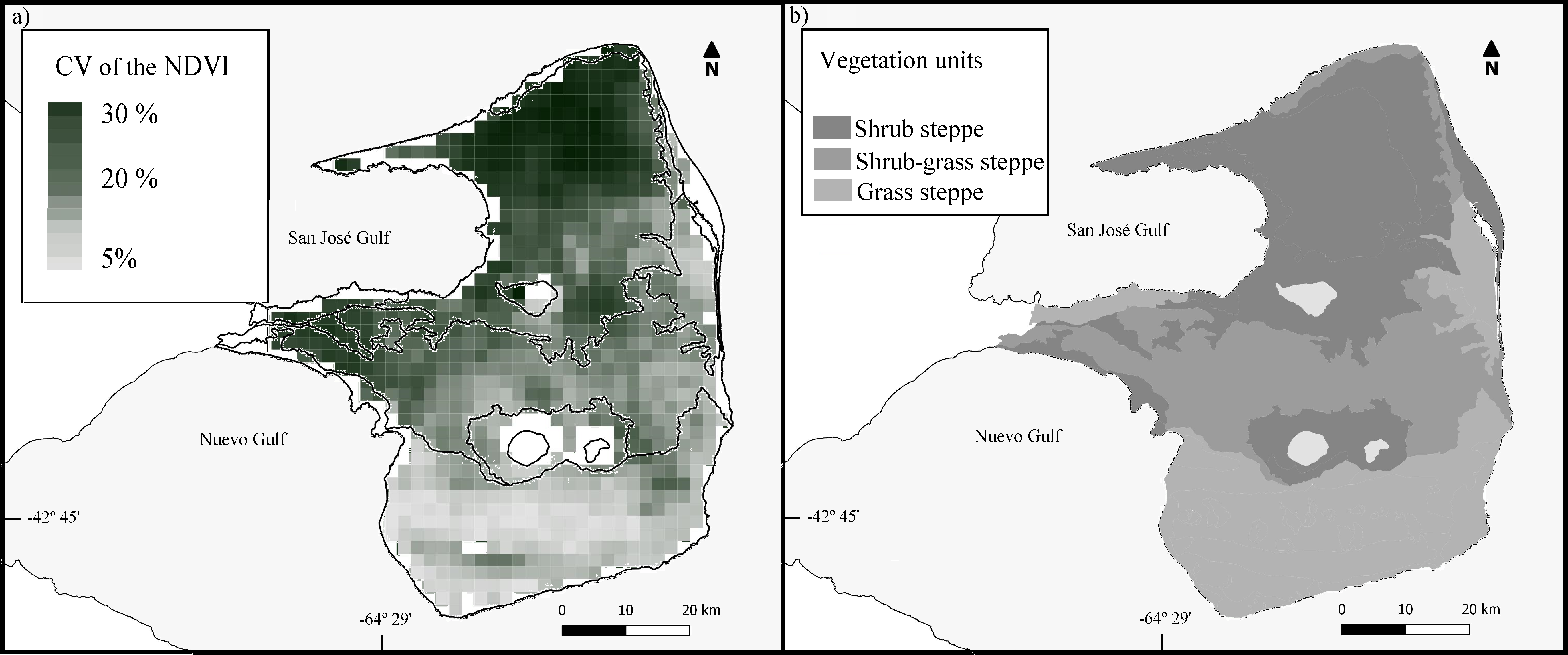


**Figure SI.2.1** a) The variation coefficient of the NDVI. The limits of vegetation units are indicated in black. b) Vegetation units of the Peninsula Valdés (obtained from Bertiller et al., 2017).

**Table SI.2.1.** The variation coefficient of the NDVI by vegetation unit. Different letters indicate significant differences (*P* < 0.005) according to the Wilcoxon rank sum test.

| Vegetation unit | Mean of the CV of the NDVI between 2010 - 2014 | Confidence interval (95%) |
| --- | --- | --- |
| Shrub steppe | 17.9^a^ | 17.61 - 18.26 |
| Grass-shrub steppe | 15.45^b^ | 15.23 - 15.67 |
| Grass steppe | 11.81^c^ | 11.46 - 12.17 |


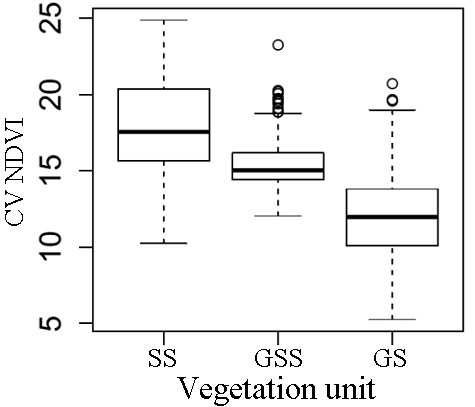


**Figure SI.2.2.** Box-plot of the variation coefficient of the Normalized Vegetation Index (CV NDVI) by each vegetation unit: Shrub steppe (SS), grass-shrub steppe (GSS) and grass steppe (GS).
