# Supplementary material for: Modeling the spatial structure of the endemic mara (Dolichotis patagonum) across modified landscapes
Source: PeerJ. 2019 Feb 12;7:e6367. doi: 10.7717/peerj.6367 (PMC6376934; doi:10.7717/peerj.6367)
Supplement: Supplemental Information 3 — As we described in the article, we evaluated concurvity measures between smooth terms throughout the model fitting procedure. Here we presented the pairwise concurvity measures by three related indices (worst, observed and estimated) for the base model of the Tweedie response distribution (Tables SI.2.1, SI.2.2 and SI.2.3), and for the final model selected (Tables SI.2.4, SI.2.5 and SI.2.6). [file peerj-07-6367-s003.docx]

**Supplemental information SI3. Concurvity measures between smooth terms**

As we described in the article, we evaluated concurvity measures between smooth terms throughout the model fitting procedure. Here we presented the pairwise concurvity measures by three related indices (worst, observed and estimated) for the base model of the Tweedie response distribution (Tables SI.3.1, SI.3.2 and SI.3.3), and for the final model selected (Tables SI.3.4, SI.3.5 and SI.3.6).

**Table SI.3.1.** Concurvity “worst measures” between each pair of univariate smooth terms of the base model with a Tweedie response distribution.

| terms | worst.  para | worst.s.  mean ndvi | worst.s.  cv ndvi | worst.s.  cv altitude | worst.s.  ranch dist. | worst.s.  sheep stock. | worst.s.  longitude |
| --- | --- | --- | --- | --- | --- | --- | --- |
| para | 1 | 4.46E-26 | 3.18E-23 | 3.50E-25 | 3.85E-24 | 2.60E-26 | 3.85E-22 |
| s(mean ndvi) | 4.49E-26 | 1 | 0.3057 | 0.1007 | 0.1155 | 0.3682 | 0.1845 |
| s(cv ndvi) | 3.18E-23 | 0.3057 | 1 | 0.1469 | 0.1064 | 0.2446 | 0.1580 |
| s(cv altude) | 3.50E-25 | 0.1007 | 0.1469 | 1 | 0.1632 | 0.1286 | 0.2067 |
| s(ranch dist.) | 3.86E-24 | 0.1155 | 0.1064 | 0.1632 | 1 | 0.4515 | 0.1986 |
| s(sheep stock) | 2.70E-26 | 0.3682 | 0.2446 | 0.1286 | 0.4515 | 1 | 0.2546 |
| s(longitude) | 3.85E-22 | 0.1845 | 0.1580 | 0.2067 | 0.1986 | 0.2546 | 1 |

**Table SI.3.2.** Concurvity “observed measures” between each pair of univariate smooth terms of the base model with a Tweedie response distribution.

| terms | observed.  para | observed.  s.mean ndvi | observed.s.  cv ndvi | observed.s.  cv altitude | observed.s.  ranch dist. | observed.s.  sheep stock. | observed.s.  longitude. |
| --- | --- | --- | --- | --- | --- | --- | --- |
| para | 1 | 2.14E-29 | 3.47E-29 | 5.90E-30 | 2.93E-31 | 2.25E-32 | 1.14E-26 |
| s(mean ndvi) | 4.49E-26 | 1 | 0.0351 | 0.0634 | 0.0513 | 0.1575 | 0.1442 |
| s(cv ndvi) | 3.18E-23 | 0.2623 | 1 | 0.0619 | 0.0632 | 0.1508 | 0.0612 |
| s(cv altude) | 3.50E-25 | 0.0436 | 0.0126 | 1 | 0.0275 | 0.0801 | 0.0319 |
| s(ranch dist.) | 3.86E-24 | 0.0529 | 0.0087 | 0.0817 | 1 | 0.0471 | 0.0323 |
| s(sheep stock) | 2.70E-26 | 0.1826 | 0.0316 | 0.0681 | 0.2905 | 1 | 0.1072 |
| s(longitude) | 3.85E-22 | 0.1250 | 0.0073 | 0.0949 | 0.0409 | 0.0431 | 1 |

**Table SI.3.3.** Concurvity “estimated measures” between each pair of univariate smooth terms of the base model with a Tweedie response distribution.

| terms | estimate.para | estimate.  s.mean ndvi. | estimate.s.  cv ndvi. | estimate.s.  cv altitude | estimate.s.  ranch dist. | estimate.s.  sheep stock. | estimate.s.  longitude. |
| --- | --- | --- | --- | --- | --- | --- | --- |
| para | 1 | 4.25E-28 | 1.71E-25 | 7.04E-28 | 1.73E-26 | 4.92E-29 | 1.48E-24 |
| s(mean ndvi) | 4.49E-26 | 1 | 0.0649 | 0.0580 | 0.0394 | 0.1438 | 0.1141 |
| s(cv ndvi) | 3.18E-23 | 0.2407 | 1 | 0.0761 | 0.0740 | 0.1705 | 0.0590 |
| s(cv altude) | 3.50E-25 | 0.0505 | 0.0389 | 1 | 0.0574 | 0.0678 | 0.0367 |
| s(ranch dist.) | 3.86E-24 | 0.0431 | 0.0161 | 0.0545 | 1 | 0.2818 | 0.0563 |
| s(sheep stock) | 2.70E-26 | 0.1892 | 0.0470 | 0.0442 | 0.3139 | 1 | 0.0845 |
| s(longitude) | 3.85E-22 | 0.1118 | 0.0235 | 0.0619 | 0.1014 | 0.1061 | 1 |

**Table SI.3.4.** Concurvity “worst measures” between each pair of univariate smooth terms of the final model selected.

| terms | worst.  para | worst.s.  ranch dist. | worst.s.  longitude |
| --- | --- | --- | --- |
| para | 1 | 3.85E-24 | 3.84E-22 |
| s(ranch dist.) | 3.86E-24 | 1 | 0.1986 |
| s(longitude) | 3.85E-22 | 0.1986 | 1 |

**Table SI.3.5.** Concurvity “observed measures” between each pair of univariate smooth terms of the final model selected

| terms | observed.  para | observed.s.  ranch dist. | observed.s.  longitude |
| --- | --- | --- | --- |
| para | 1 | 5.7E-31 | 1.10E-26 |
| s(ranch dist.) | 3.86E-24 | 1 | 0.0318 |
| s(longitude) | 3.85E-22 | 0.0411 | 1 |

**Table SI.3.6.** Concurvity “estimated measures” between each pair of univariate smooth terms of the final model selected.

| terms | estimated.  para | estimated.s.  ranch dist. | estimated.s.  longitude |
| --- | --- | --- | --- |
| para | 1 | 1.73E-26 | 1.48E-24 |
| s(ranch dist) | 3.86E-24 | 1 | 0.0563 |
| s(longitude) | 3.85E-22 | 0.1014 | 1 |
