# Supplementary material for: Modeling the spatial structure of the endemic mara (Dolichotis patagonum) across modified landscapes
Source: PeerJ. 2019 Feb 12;7:e6367. doi: 10.7717/peerj.6367 (PMC6376934; doi:10.7717/peerj.6367)
Supplement: Supplemental Information 4 — Spatial autocorrelation in the residuals was evaluated using the ‘dsm.cor’ function of the‘dsm’ package. As described in the article, the correlogram show a small amount of spatial autocorrelation in the residuals (Fig. SI3.1). The confidence interval increased in width as the number of lags increased. [file peerj-07-6367-s004.docx]

**Supplemental information SI4. Spatial autocorrelation in the residuals**

Spatial autocorrelation in the residuals was evaluated using the ‘dsm.cor’ function of the‘dsm’ package. As described in the article, the correlogram show a small amount of spatial autocorrelation in the residuals (Fig. SI4.1). The confidence interval increased in width as the number of lags increased.


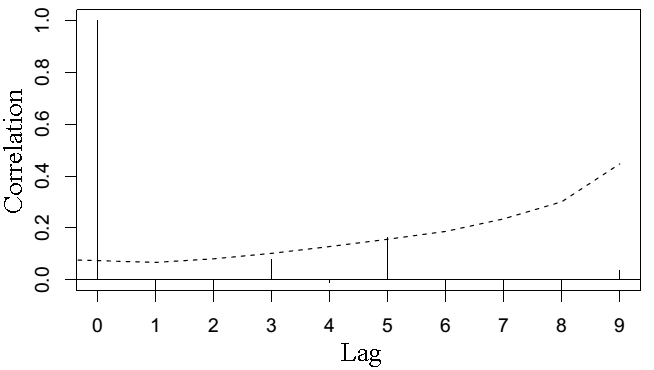


**Figure SI.4.1.** Autocorrelation of deviance residuals between segments (lags) for the fitted density surface model selected. The dashed line represents the 95% confidence interval. Lag 0 is the correlation between a segment and itself, Lag 1 between a segment and its immediate neighbours (i.e. segments that touch), Lag 2 between a segment and the segment one segment away, and so on. Correlations are only calculated within a given transect.
