# Supplementary material for: Modeling the spatial structure of the endemic mara (Dolichotis patagonum) across modified landscapes
Source: PeerJ. 2019 Feb 12;7:e6367. doi: 10.7717/peerj.6367 (PMC6376934; doi:10.7717/peerj.6367)
Supplement: Supplemental Information 6 — This file indicates the link that explained the modelling procedure that was implemented in this study. [file peerj-07-6367-s006.docx]

**Supplemental information SI5. Modeling procedure**

The modelling procedure that was implemented with the dsm package in an R environment, can be checked in http://github.com/DistanceDevelopment/dsm
